# O19 A Complex Case of Systemic Lupus Erythematosus with Class IV Lupus Nephritis in a 15-year-old Egyptian Girl

**DOI:** 10.1093/rap/rkaa054.007

**Published:** 2020-11-03

**Authors:** Dr Saadia Malik, Dr Ahlam Al Ynbiawi, Dr Fadi Mourad

**Affiliations:** Dr Sulaiman Al Habib Medical Group, Riyadh, Saudi Arabia

## Abstract

**Case report - Introduction:**

We present a case of 15-year-old girl referred to rheumatology clinic by Infectious disease consultant in a private hospital. Her presentation included fever, inflammatory joint pains, butterfly rash and finger pulp infarcts. Her immunology showed highly positive anti DsDNA (>1000), positive anti smith and anti Ro antibodies. This case eludes challenges of management as she went on to develop lupus nephritis. She was treated with IV Methylprednisolone pulse therapy followed by tapering course of steroids, Mycophenolate Mofetil and Hydroxychloroquine. The immunosuppressive treatment including cyclophosphamide and Rituximab could not be given due to lack of approval by her insurance provider.

**Case report - Case description:**

This 15-yearold Egyptian young girl presented initially in September 2019 to Emergency department with fever and facial rash. This episode was treated as allergic urticaria. Subsequently, she was referred to see the Infectious disease consultant who requested multiple investigations including blood culture, anti DsDNA and ENA antibodies.

She presented to Rheumatology clinic with malar rash, mouth ulcers, finger pulp infarcts and joint pains. Her Immunology showed ANA 1:640, Anti DsDNA 1031, Anti Ro 27, Anti Sm 30, Anti nRNP/Sm 28 and elevated Urinary Albumin/Creatinine ratio (27 mg/mmol). A diagnosis of Systemic Lupus Erythematosus with Lupus Nephritis was made. She was initiated on tapering course of steroids (30 mg starting dose) and Plaquanil 400 mg. The follow up visit confirmed improvement (including facial rash) and urine ACR of 17 mg/mmol. The steroids were tapered by 5 mg every 2 weeks and Azathioprine was considered.

Unfortunately, she developed renal flare in December 2019 which coincided with reduction in steroid. Urine ACR value 383 mg/mmol, ESR 110 and Anti DsDNA was >1000. The request to offer induction therapy with Rituximab (as she was not keen for Cyclophosphamide) did not receive approval by her insurance company. Her case was reviewed by nephrologist, biopsy was not done at this stage. Her condition was managed with Mycophenolate and Pulse steroid therapy.

In February 2020, she had further renal flare (Urine ACR 694 mg/mmol) and that required increase in the dose of steroids. Unfortunately, there was still disapproval by insurance company despite multiple requests. Over the course of next 3 months, there was poor compliance with clinic attendance coinciding with COVID-19pandemic. Her medication included oral Prednisolone 15 mg OD, Mycophenolate 3 gm and Hydroxychloroquine 400 mg.

More recently, she has had significant renal flare treated under Nephrologist with Pulse therapy of IV Methylprednisolone with lack of approval to give Rituximab by her insurance provider.

**Case report - Discussion:**

This is a challenging case of Lupus nephritis in a young adolescent girl seen in a private hospital in Riyadh, Kingdom of Saudi Arabia.

She was concerned about her appearance in her first presentation due to disfiguring facial rash. This rash completely resolved, and patient was very satisfied to the extent of some lack of engagement in follow up subsequently.

This clinic is based in a busy and popular private hospital in Riyadh where non-Arabic speaking clinician rely on translator to communicate with patient and this can make the consultation further challenging. The clinician must make sure, in brief consultation time, to get the exact message across. My experience did teach me that there were certain times when medications were not taken as intended due to lack of full understanding by the patient.

In a private hospital setting, patient can choose their own compliance with clinic attendance as per their convenience and understanding of the condition. This aspect also contributed towards management challenges and control of her disease.

The most limiting and frustrating factor to manage this case has also been the lack of engagement and approval by the insurance provider. I made multiple medical requests for their attention and unfortunately all were rejected.

The local healthcare system in Kingdom of Saudi Arabia allows expat patients to be seen primarily in private hospitals under the cover of their employer insurance. This also led to lack of sharing expertise and treatment facilities with the regional specialist rheumatology centre, which is mainly designed to cater for local nationals. I am seriously considering making a “special request” for this case to be accepted in the Ministry hospital so Cyclophosphamide and/or Rituximab can be considered.

**Case report - Key learning points**
 

1. Should we have considered Mycophenolate at very initial presentation and could that have led to different and better clinical outcome?
2. Would renal biopsy change management significantly and how best we can persuade patient and Nephrologist?
3. How else we can engage this young adolescent girl as she was more concerned about her cosmetic appearance and since skin has responded very well, she may not feel too convinced on the need for ongoing treatment and also attending clinic appointment?
4. Perhaps we could have referred her case as a “special request” to local specialist Rheumatology center so she could have been considered for treatment options like Rituximab and cyclophosphamide. The local healthcare in Saudi Arabia permits local nationals to be seen in local and private hospitals whilst expats are catered only in private hospital unless there is exception due to availability of treatment or expertise, where process of acceptance can also be complex and time demanding.

